# Renovation of Agro-Waste for Sustainable Food Packaging: A Review

**DOI:** 10.3390/polym15030648

**Published:** 2023-01-27

**Authors:** Sandhya Alice Varghese, Harikrishnan Pulikkalparambil, Khwanchat Promhuad, Atcharawan Srisa, Yeyen Laorenza, Lerpong Jarupan, Tarinee Nampitch, Vanee Chonhenchob, Nathdanai Harnkarnsujarit

**Affiliations:** 1 Department of Packaging and Materials Technology, Faculty of Agro-Industry, Kasetsart University, 50 Ngam Wong Wan Rd., Latyao, Chatuchak, Bangkok 10900, Thailand; 2 Center for Advanced Studies for Agriculture and Food, Kasetsart University, 50 Ngam Wong Wan Rd., Latyao, Chatuchak, Bangkok 10900, Thailand

**Keywords:** agro-waste, biopolymer, food packaging, residue

## Abstract

Waste management in the agricultural sector has become a major concern. Increased food production to satisfy the surge in population has resulted in the generation of large volumes of solid waste. Agro-waste is a rich source of biocompounds with high potential as a raw material for food packaging. Utilization of agro-waste supports the goal of sustainable development in a circular economy. This paper reviews recent trends and the development of agro-wastes from plant and animal sources into eco-friendly food packaging systems. Different plant and animal sources and their potential development into packaging are discussed, including crop residues, process residues, vegetable and fruit wastes, and animal-derived wastes. A comprehensive analysis of the properties and production methods of these packages is presented. Future aspects of agro-waste packaging systems and the inherent production problems are addressed.

## 1. Introduction

**Figure 1:**
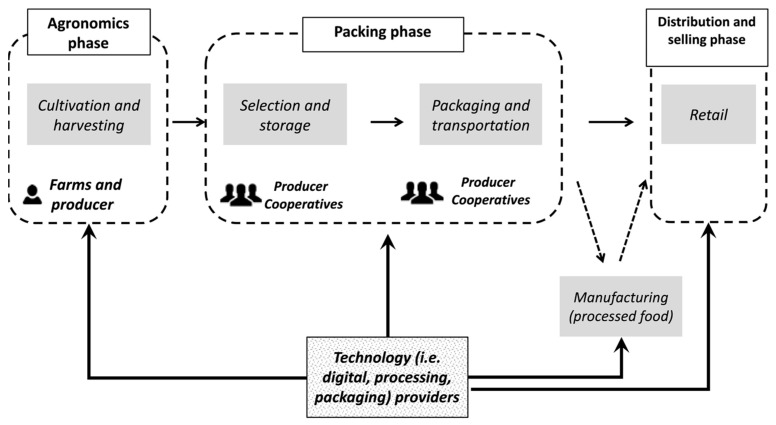
Food supply chain (Reproduced with permission from Ciccullo et al. (2021) [5].

Globally, food loss as waste accounts for nearly one-third of total production, leading to reduced economic benefits and environmental pollution [1]. Food loss and waste generated as worthless exhaustion of food resources are challenges to a robust sustainable food chain ecosystem. Generation of greenhouse gases in food production also pollutes the environment [2]. The Food and Agricultural Organization (FAO) defines food loss and wastage differently. Food loss is the reduction in mass or quality of food initially produced for consumption. These losses occur across different stages of the food supply chain, including production, handling and storage, and processing, while food waste is the amount of food discarded either due to mismanagement or spoilage. Food waste mainly occurs during the distribution and consumption stages of the food supply chain [3,4]. Currently, research in the food industry focuses on minimizing food loss and waste in a holistic matter to balance food quality. The utilization of food waste has been taken up as a challenge to achieve sustainable development through a circular economy. The three approaches suggested to minimize food loss and waste are to reduce, reuse, and recycle. Figure 1 shows the different stages in the food supply chain.

Harvested agro-produce must be properly handled, processed, packed, and stored, especially during transportation. Appropriate protocols should be followed to prevent food loss and waste throughout the supply chain [6]. Packaging, especially conventional plastic packaging, is a large source of waste and environmental pollution. Appropriate packaging should serve four main functions for food applications, namely protection, containment, convenience, and communication, which are essential to ensure quality and safety in food consumption [7]. A wide variety of materials is used as packaging. Recent developments in packaging technology include the evolution of active and smart packaging, where active antioxidant, antimicrobial, flavoring, and oxygen-scavenging agents are added to supplement the desired packaging characteristics and enhance shelf life [8,9,10,11,12,13]. However, increasing concerns have been expressed about the use of fossil-based plastic packages for food coverings, which negatively impact the environment [14]. Therefore, alternative eco-friendly packaging materials are now preferred by both global consumers and the packaging industry.

Eco-friendly packages can serve the purpose of sustainable food protection [15]. Food packaging is mainly utilized as a single-usage wrap and this triggers the waste generated by the plastic packaging industry. Bioplastics are sustainable materials for the packaging industry [16,17,18]. However, these environmentally friendly plastics have certain drawbacks. The biodegradability of bioplastics is affected by temperature and most end up in landfill mixed with other recyclable plastics, thereby affecting the recycling system structure. Bioplastics are also relatively costly, which limits their application, and they have limited mechanical and barrier properties [19,20]. Therefore, packaging made from bioresources requires the input of modern technology to improve the required properties.

The increased environmental concern of global consumers has driven the research and development of alternative packaging materials. Numerous biomaterials derived from bioresources have shown potential for use as packaging. Agro-waste is a rich source of biopolymers and bioactive compounds. Extraction and purification of these materials as alternative packaging boosts sustainable development. This review discusses the potential utilization of agro-waste as packaging. Findings suggest that agro-waste can be utilized for food packaging, thereby supporting sustainable development goals.

## 2. Industrial Agro-Waste

**Figure 2:**
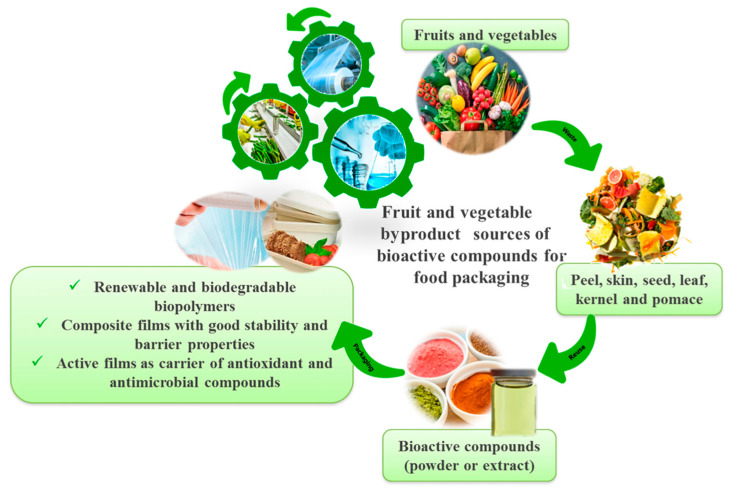
Potential application of agro-waste products in packaging [27].

In a developing economy, rapid population growth and urbanization result in product shortages, unable to meet growing consumer demand. This calls for low-priced energy sources using cost-effective agricultural residues [21]. These include unusable agricultural by-products such as crop residues, process residues, vegetable and fruit waste and peels, and animal waste, including poultry by-products and dairy products. Most of these wastes end up as liquids, slurries, or solids in landfills. Agro-industrial and other solid waste disposal have now become a critical concern, along with the food wastage and loss discussed earlier [22]. The waste derived from natural sources mainly contains carbon atoms that can potentially be used as a source of carbon material such as wood for thick carbon electrodes [23], soybean protein isolates as a source of porous carbon sheet [24], and matamba fruit shell waste as a source of biochar [25]. Furthermore, the incorporation of amorphous carbon powder in food packaging exhibits bactericidal properties due to its high oxidoreductive potential, resulting in cell membrane damage during lipid peroxidation and intensification of oxidative stress in microbes, which can potentially be used in active packaging application [26]. Types of food packaging systems developed using agro-waste are shown in Figure 2.

### 2.1. Agro-Waste Generation

**Figure 3:**
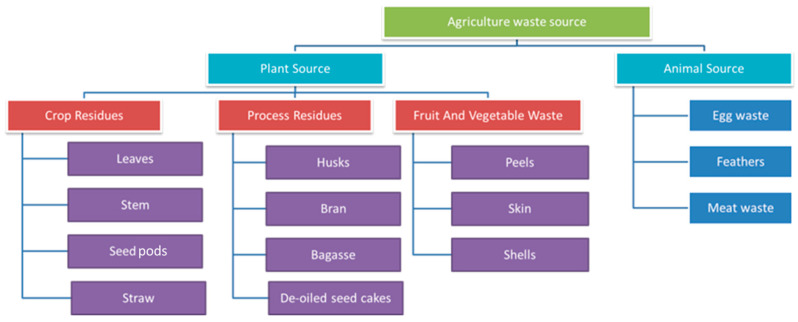
Various sources of agricultural waste.

Agricultural waste, or agro-waste as it is generally known, is the leftovers derived from the generation and processing of various agro-products, including fruits, vegetables, meat, and dairy products [28]. Agricultural wastes emanate from both plant and animal sources. A detailed classification of these wastes is shown in Figure 3.

Rapid urbanization and population growth as well as industrial growth have generated vast quantities of solid waste that have now become a major social problem. Annual generation of agro-waste has mushroomed, and without proper disposal methods this causes environmental pollution as well as health hazards for both humans and animals. Most agro-industrial wastes are rich in bio-compounds that are poorly treated and underutilized. Over one billion tons of solid waste are generated annually, polluting the environment and producing socioeconomic ill effects. This is a wake-up call for the proper management of an enormous amount of waste. Researchers are actively seeking new, innovative, and eco-friendly technologies for waste management [29,30]. With its huge availability and potential economic value, agro-waste utilization is now considered an important aspect of sustainability [31].

The waste that could be utilized in the packaging material field must be categorized as non-hazardous solid waste for further safe usage. Waste management, including recycling and utilization, has been regulated by the Resource Conservation and Recovery Act (RCRA) under the Environmental Protection Agency (EPA, US) [32]; they have specified the recycled materials that must be excluded from solid waste in processing; (i) waste used as an ingredient; (ii) waste used as a product substitute; and (iii) waste returned to the production process. Waste can be used as an ingredient if the waste was directly included in the ingredient in the production process. Waste can be used as a product substitute when the material is directly used to replace an existing product without first being reclaimed, while wastes can be returned to the production process when the material is returned to the production process as a feedstock or as raw material. Agricultural waste is one of the excluded hazardous wastes and is defined as a solid waste generated by the growing and harvesting of agricultural crops or the raising of animals, including animal manures, which are returned to the soil as fertilizers [32].

Agro-waste has recently been intensively studied, generating a wide range of valuable materials including biofuels, enzymes, vitamins, antioxidants, animal feed, antibiotics, and other chemicals. Usage of agro-waste as a potential base material for sustainable food packaging is discussed in the following sections.

### 2.2. Composition of Agricultural Waste Residues in Packaging

The utilization of agro-mass has recently attracted great interest. The composition of agro-waste is a major factor when considering possible utilization. Agricultural residues are a significant source of raw materials that can produce goods with additional value. Most agricultural wastes are lignocellulosic materials that mainly consist of cellulose (35–50%), hemicellulose (20–35%), lignin (15–25%), and a number of other chemicals. The compositions of common agro-wastes are shown in Table 1 [33,34].

## 3. Packaging Developments Using Agro-Waste

Materials derived from agro-waste such as cellulose, hemicellulose, chitin, lignin, starch, pectin, alginate, and protein have the potential to be turned into food packaging [35], with several advantages such as non-toxicity, biodegradability, wide availability, and biocompatibility with other materials, which can improve the quality of the packaged materials and extend shelf life [36]. Solid waste originating from crop residues, process waste, and animal by-products has been extensively applied as a filler or main ingredient in packaging. The properties and the interactions between these materials and other matrixes are interesting to explore.

### 3.1. Utilization of Crop Residues in Packaging

Various crop parts, including the leaf, stem, seed pod, and straw, have been used to generate packaging material (Table 2), with high potential for valorization and development into eco-friendly packaging to reduce waste generation. In India, serving food on leaf plates is a custom with cultural, religious, and socioeconomic impacts [37]. The processing of cereal, sugarcane, and dairy products produces residues and by-products that can be used economically. By serving as a substrate for edible, biodegradable, antibacterial, antioxidant, and agricultural films or coatings with multiple other functional features, a variety of by-products, wastes, and residues have the potential to be valued and reduce waste. After the grains from crops such as wheat, rice, maize, barley, etc., are harvested, residues in the shape of stems and stalks (stubble) are produced. The residues are used as animal feed or left in the ground to prevent erosion, but these approaches are less efficient and may encourage the spread of unwelcome pests. Burning residues causes significant air pollution and nutrient loss, which have detrimental effects on the environment and human health. Therefore, the best option is to use these residues as a significant supply of fibrous biomass, which has the potential to be used economically to produce paper and bioplastics. Some agricultural waste containing lignocellulose can be degraded by fungi that have a filamentous network called mycelium, which mainly contains chitin, glucan, protein, and lipids [38]. Using agricultural waste as a source for bioplastic manufacture helps to solve disposal issues, minimize environmental pollution, use less petroleum-based synthetic polymers, and creates biodegradable plastics at a far lower cost.

Dissanayake et al. (2021) [39] reported the use of areca nut leaf sheaths to fabricate transparent packaging material with mechanical properties comparable to polyethylene. Bleaching and coating the leaf sheaths with carboxy methylcellulose were non-toxic processes and the developed packaging system was environmentally friendly. Stem extracts of many plants have been used to develop active food packaging systems. Kaya et al. (2018) [40] developed chitosan-based antimicrobial packaging film incorporating stem, leaf, and seed extracts of *Pistacia terebinthus*. Methylcellulose films with ginja cherry stem extract were found to have anti-bacterial properties and UV resistance by Campos et al. (2014) [41] as an effective way to utilize an otherwise waste product. Superabsorbent aerogels were developed from cellulose extracts based on *A. donax* stem biomass by Candia et al. (2019) [42]. These aerogels served as bioactive food packaging pads for refrigerated meat storage. Crop straw has been extensively studied as a reinforcing material in biocomposite food packaging films. Three different kinds of wheat straw fibers were incorporated in PHBV-based sustainable food packaging films by Berthet et al. (2015) [43]. They found that increasing the fiber size decreased tensile strength while increasing the water vapor transmission rate, which is advantageous in the packaging of respiring fruits. Studies on shrimp waste-derived chitosan film containing rice straw were conducted by Elhussieny et al. (2020) [44]. Results suggested that the addition of rice straw nanofibers improved the mechanical properties of the films and demonstrated compatibility for food packaging applications. Cellulose extracted from crop waste has also been used in biocomposite preparation. González et al. (2019) [45] used compression molding to incorporate cellulose extracted from *Posidonia oceanica* leaf biomass as a filler in corn starch films at up to 40 wt% loading. The films showed improved mechanical and moisture barrier properties and enhanced storage stability.

Kumar et al. (2023) [46] studied the main component used to create paper-based packaging material, lignocellulosic mass, which is present in significant amounts in pine needles and pinecone trash. Plastics were eliminated from packaging using plant waste as a packaging material, which also supports a circular economy. They created ethylene-scavenging paper using waste pine needles combined with micro-fibrillated cellulose and halloysite nanotubes. Tran et al. (2022) [47] developed CO2-based biocomposites, which they devised to valorize vegetable wastes as useful and affordable natural antioxidant resources. Dried parsley and spinach stems were firstly micronized. Using the hot compression molding method, dried vegetable stems were incorporated into a poly (propylene carbonate) (PPC) polymer matrix. Olawuyi and Lee (2022) [48] demonstrated mucilage polysaccharides and carboxymethyl cellulose isolated from okra leafstalk wastes that were then used to create polysaccharide-based composite films. Therefore, new bio-packaging films with a special combination of properties are necessary to ensure the bio-packaging films’ best qualities and eliminate synthetic plastics. Lima et al. (2021) [49] presented a project to create a rigid packaging biocomposite based on PLA and mango by-products. Extrusion/injection processing was used to create six biocomposites employing formulations with PLA as a matrix and up to 20% by weight of mango seed byproducts. Thivya et al. (2021) [50] focused on the utilization of selected waste streams such as shallot stalks and tamarind seeds for the development of packaging films that can be used as an alternative to plastic films due to the increased generation of agricultural bio-waste, particularly onion solid waste, and its impact on the environment. Rincón et al. (2021) [51] demonstrated sequential subcritical water extraction utilizing various time and temperature combinations to investigate the possible separation of bio-active polysaccharides from bay tree pruning waste. The extracted polysaccharides had optimal characteristics for use as an additive in food packaging, since they were significantly pectin-enriched, while maintaining their high molecular mass (10–100 kDa). Elhussieny et al. (2020) [44] developed composite films made of rice straw and rice straw nanofibers as reinforcements and chitosan in polymeric matrices. Wet mixing and casting processes were used to create the films, and different reinforcement amounts (25–35 wt%) were used. The purpose of this study was to explore the synthetic composite films’ physical, mechanical, chemical, and thermal properties to assess whether they could be used to package food or not. Bilo et al. (2018) [52] produced a new bioplastic created from rice straw, a type of agricultural waste that is typically not recovered. The sample was prepared for synthesis using the Naviglio extractor before being dissolved in trifluoroacetic acid. Kasaai and Moosavi (2017) [53] utilized mandarin waste extracts to enhance Kraft paper’s water and gas barriers as the main goal of their investigation.

**Table 2:** Packaging developments utilizing crop residues.

Waste Source	Components	Extraction	Contents of Waste in Packaging	Polymer Blend	Role of Waste in Packaging	Packaging Form	Production Process	Major Findings	Ref.
Pine needles	Cellulose	Sodium hydroxide (NaOH) and sodium sulfide (Na_2_S)	65–90%	Microfibrillated cellulose (MFC) and halloysite nanotubes (Hal)	Matrix	Paper	Sheet former	Presence of Hal made the paper an oxygen scavenger and also improved mechanical properties.	[46]
Pine, eucalyptus, corn straw	Nanofiber	Sodium hydroxide extraction	>30%	Starch from rice flour	Filler	Film	Solution casting	The higher the nanofiber concentration, the lower the water solubility and water vapor permeability and the higher the opacity of the films.	[54]
Parsley and spinach stems	Powder	2,2′-azinobis(3-ethylbenzothiazoline-6-sulfonic acid) diammonium salt (ABTS)	30–70 wt%	Poly (propylene carbonate)	Antioxidant	Film	Hot compression molding	Improved mechanical properties and water vapor permeability due to strong intermolecular hydrogen bond network between matrix and agro-waste. Good antioxidant activity characterized by scavenging of ABTS•+ cationic radical.	[47]
Okra leaf stalk	Cellulose	Alkaline deep eutectic solvent	70–100%	Mucilage polysaccharides	Matrix	Film	Solution casting	With increasing mucilage content up to 10%, oxygen permeability, water barrier properties, and antioxidant activity improved due to the compact network of hydrogen bonding. As a result, cherry tomatoes were effectively preserved for 14 days of storage.	[48]
Mango seed	Fiber	Sodium hypochlorite extraction	10–20%	PLA	Reinforcement	Sheet	Injection molded	Mechanical and barrier properties improved with 20 wt% fiber addition which increased elastic modulus up to 38%.	[49]
Tamarind seed	Xyloglucan as powder	Microwave assisted extraction	2 and 4%	Shallot stalk powder	Matrix	Film	Solution casting	Good light barrier, antioxidant properties due to polyphenol–biopolymer interactions. Adequate elongation at break due to flexible molecular structure of xyloglucan.	[50]
Bay tree prunings	Carbohydrate	Water extraction	10–40%	Chitosan	Filler	Film	Solution casting	Film with 10% addition showed good tensile properties, while film with 20% addition had good barrier properties. Antimicrobial activity was independent of the filler and due to interaction between positively charged amino groups in chitosan and negatively charged microbial membranes, leading to cell disruption.	[51]
Chinese chive root	Extract	Ethanol extraction	1–5%	Chitosan	Active agent	Film	Solution casting	Barrier properties improved due to the cross-linking effect between the matrix and additive. Mechanical properties reduced because the additive disturbed the crystalline structure and inhibited inter-chain interaction of the matrix. Good antioxidant and antibacterial properties owing to the presence of phenolic groups.	[55]
Rice straw	Cellulose fiber	Sodium hydroxide extraction	25 and 35%	Chitosan	Reinforcement	Film	Solution casting	Addition of 25 wt% had the highest mechanical property due to adequate stress transfer between the fiber and the matrix and formation of three-dimensional networks.	[44]
Rice straw	Cellulose	Naviglio extractor	100%	-	Matrix	Film	Solution casting	Good mechanical properties, tear resistance and flexibility. Dual shape memory effect. Completely degradable after 105 days of burial.	[52]
Mandarin leaves and peel	Cellulose	Hexane and chloroform extraction	100%	-	Matrix	Paper	Kraft paper	Improved water and gas barriers of Kraft paper.	[53]

### 3.2. Utilizations of Process Waste in Packaging

The main solid wastes generated in the production cycle of crops include straw, husk, ash, bran, and bagasse. Various studies have reported the use of these waste materials to fabricate sustainable food packaging (Table 3). The utilization of agricultural and food industry residues as a rich source of biopolymers such as cellulose, starch, fiber, and protein can be obtained by mechanical (milling [56,57,58]) and chemical (ethanol extraction [59], acid extraction [60], alkaline extraction [61]) treatments. Using reinforcements obtained from agricultural waste becomes of greater interest when used to enhance the properties of bio-based food packaging materials in terms of mechanical and thermal resistance, water absorption, and barrier and biodegradation properties [61,62,63,64].

Using bio-based reinforcement material as antioxidant compounds derived from food and agricultural by-products is a subject of research for use in active bio-based food packaging. Rodríguez-Félix et al. (2022) [65] developed zein films with ultrafiltered betalain extracted from beet bagasse, which showed the most hydrophobicity, higher antioxidant activity, and higher amount of betalains (specifically betacyanins). In contrast, Tkaczewska et al. (2023) [59] found that single- and double-layer edible films based on soybean protein hydrolysates and furcellaran enriched with ethanol extract from soy husks were not efficient in inhibiting the oxidation of tofu lipids, and tofu had higher weight loss as well as hardness during the storage period compared to the control. Thus, the differences between extraction techniques, antioxidants, or phenolic compounds remaining in the solid residues after such extractions and interaction between the antioxidants and the polymer matrix are possible factors that influence the antioxidant properties of materials when applied to food products.

Moustafa et al. (2020) [66] studied rice bran with tea tree oil incorporated into low-density polyethylene-grafted acrylic acid. Results revealed that the mechanical properties were unaffected by the addition of filler to polyethylene. Enhancement in hardness and water vapor barrier properties was observed up to 30 wt% filler addition. The antimicrobial efficiency of these films suggested that they were suitable candidates for food packaging applications. Bascón-Villegas et al. (2022) [67] incorporated lignocellulose nanofibers from wheat straw waste at a maximum of 1% in PLA–PBAT blends to develop biocomposite films on a pilot scale. Results showed improved optical characteristics, water vapor permeability, and antioxidant and antimicrobial properties for the agro-waste added films, with comparable results to commercial packaging for fresh-cut lettuce. Kumar et al. (2019) [68] isolated carboxy methylcellulose (CMC) from rice husks and sugarcane bagasse. The CMC was then blended with starch at a ratio of 1:1 to prepare a biofilm via solution casting. The blend film showed promise for use in food packaging due to its improved flexibility, adequate opacity, and mechanical properties. Menzel et al. (2020) [69] fabricated starch films with an aqueous extract of rice straw that was rich in phenolic antioxidant compounds. The films showed improved oxygen barrier properties, but transparency was reduced and the films were more brittle. The biofilms were suggested as a packaging material for food products such as nuts to retard lipid oxidation. In a similar study, Freitas et al. (2022) [70] developed PLA films containing up to 6% aqueous extract of rice straw using the compression molding technique. The films they developed showed decreased tensile and barrier properties but improved antioxidant capacity and could be used to preserve aqueous as well as fatty food products.

High moisture absorption, hydrophilia, and permeability are major drawbacks in many bio-based materials [57,63]. Lignocellulose is a complex matrix consisting of cellulose, hemicellulose, and lignin [71]. Lignocellulose reinforcements produced from cassava bagasse [72], vegetable processing wastes [67], and cellulose fillers extracted from sugarcane bagasse showed a significant decrease in the water vapor permeability of bio-based film. The reduction in permeability of bioplastic film is associated with a network formed between the polymer matrix and reinforcement via hydrogen bonds, increasing a tortuous path and reducing the availability of hydrophilic hydroxyl groups to interact with water molecules, which makes it more difficult for the water molecules to absorb and pass through [63,69,72]. In contrast, reinforced polymer composites made using lignocellulose from raw wheat bran [67] and cellulose from rice husk flour [61] as fillers have an increase in water absorption and water vapor permeability, respectively, due to the hydrophilic properties of the fillers and the low chemical affinity between fillers and biopolymer matrices [61,67]. The incorporation of a small amount of fibers as reinforcement or filler in the bioplastic composite could reduce water absorption and water vapor diffusivity in the composite. Bioplastic film composed of 75% cellulose and 25% fiber extracted from cocoa pod husks and sugar-cane bagasse, respectively [66], and 9% cassava bagasse fibers [68] exhibited the lowest water absorption and water vapor permeability as compared with the others. This could be due to the strong interfacial bonding between the reinforcement fiber and polymer matrix, which hinders the penetration of water molecules through the matrix [61,73,74]. On the other hand, the high amount of fiber content could be creating poor interfacial bonding to the composite matrix, contributing to the formation of a more spaced structure and pores, resulting in greater water absorption and more penetration of water into the matrix [57,75].

The mechanical properties of biomaterials are also highly important for their practical applications in food preservation and packaging. Bio-composite materials with robust mechanical properties (high tensile strength and elastic modulus) can be obtained with an addition of lignocellulose nanofibers [72], cellulose nanocrystals [60], and fiber [57,63,75], yielding stiffer, more rigid, and harder bio-based packaging materials. However, the agglomeration of lignocellulose [61] and sweet potato residue fillers [58] in bioplastic leads to a breakdown of the interaction of the two matrices that results in a weak interphase adhesion and loss in mechanical properties. Thus, mechanical properties are largely improved due to (i) the stiffness of fillers, (ii) the strong hydrogen bonding between the reactive hydroxyl groups of the fillers and the polymer matrix, and (iii) the compatibility and homogeneous dispersion of fillers in the polymeric matrix [63,64].

Thermal degradation can lead to other properties (i.e., mechanical strength and barrier properties) gradually deteriorating. Lignocellulose nanofibers from eggplant crop residues obtained by mechanical pre-treatment enhanced the degradation temperature of poly (vinyl alcohol) film, which provides better thermal stability to the reinforced composites due to the major presence of lignin establishing covalent bonds with cellulose [64]. Starch and cellulose fillers from sweet potato residues showed decreases in both the decomposition temperature and the degradation rate of poly(3-hydroxybutyrate-co-3-hydroxyvalerate) (PHBV) composites. Furthermore, the thermal stability of polymer matrix composites was improved by increasing the fiber, which decreased the weight loss and decomposition rate of polymer molecules within the composite matrix [63]. Cruz-Tirado et al. (2019) [57] proved that the addition of sugarcane bagasse and asparagus peel fibers improved the thermal stability of sweet potato starch-based foam trays, which provided more stable bonds between starch and glycerol. Thus, fiber from agricultural and food residues represents one of the most promising candidates to be used as reinforcement that could provide improved thermal, mechanical, and barrier properties to biopolymer matrix composites.

**Table 3:** Packaging utilizing process residues.

Waste Source	Components	Extraction	Contents of Waste in Packaging	Polymer Blend	Role of Waste in Packaging	Packaging Form	Production Process	Major Findings	Ref.
Cocoa pod husk	Cellulose	Alkaline treatment	25–100%	Fiber extraction from sugarcane bagasse	Film-forming matrix	Film	Solution casting	Optimal combination was 75% cellulose and 25% fiber with lowest water absorption and water vapor permeability.	[61]
Rice husk flakes	Rice husk flour	Mechanical grinding and sieving	10%	Poly(3-hydroxybutyrate) (PHB) and poly(3-hydroxybutyrate-co-3-hydroxyvalerate) (PHBV)	Filler	Film	Thermo-compression	Best miscibility at 5–10 wt%. Incorporation of up to 20 wt% of rice husk produced films with high contact transparency, relatively low crystallinity, high thermal stability, improved mechanical ductility, and medium barrier performance to water vapor and aroma.	[56]
Soybean bran	Protein hydrolysates and soybean bran extract	Ethanol extraction	2.5 g furcellaran and 5 g protein hydrolysates	Furcellaran	Antioxidant compounds	Single- and double-layer films	Solution casting	Complete biodegradation within 10 days and did not demonstrate any toxicity for cress seeds during their growth. No antibacterial and antioxidant properties in tofu samples.	[59]
Raw wheat bran flakes	Lignocellulosic fillers	Milling	10, 30 and 50%	Poly (butylene succinate) (PBS)	Filler	Pellets and moldings	Twin-screw extrusion and injection moldings	Increase in degree of crystallinity with aging was as high as 48% in the composition with 10% bran content. The aging hardness of the biocomposite increased by up to 12%, and surface roughness increased by as much as 2.4 µm at the highest bran content. The weight of samples made from PBS alone after 70 days of composting decreased by 4.5%, while the biocomposite with 10% bran content decreased by 15.1% and with 50% bran by as much as 68.3%.	[62]
Beetroot bagasse	Betalain extract	Ultrafiltered extract	1, 2, and 3%	Zein	Antioxidant compounds	Film	Solution casting	Higher antioxidant activity.	[61]
Cassava bagasse	Fiber	-	3, 6, and 9%	Cornstarch	Hybridized agent and reinforcement	Film	Solution casting	Enhanced mechanical properties. Faster biodegradation and lower water absorption capacity.	[63]
Cassava bagasse	Lignocellulose nanofibers	Enzymatic pretreatments and colloidal mill	0.65 and 1.3%	Cassava starch	Reinforcement	Film	Solution casting	Opacity and water absorption values of films reduced significantly and tensile stress improved. Water vapor permeability reduced.	[72]
Cassava bagasse	Dried cassava bagasse	Dehydration and grinding	17, 33, 50, 66, and 100%	Cassava starch, gelatin, and *Spirulina platensis* biomass	Reinforcement	Film	Solution casting	The color, viscosity, solubility, thickness and moisture of the films were affected. Swelling increased during a period of 60 min, with the highest values recorded in acid medium. Maximum elongation value was found in biobased films that had 66% of *S. platensis*, 17% of cassava bagasse, and 17% gelatin.	[75]
Sugarcane bagasse and asparagus peel	Fiber	Heat drying and milling	5, 10, 15, 20, 30, and 40%	Sweet potato starch	Reinforcement	Foam trays	Thermo-pressing	Trays were less porous, with lower water absorption capacity and greater tensile strength than with addition of asparagus peel fibers. Higher concentrations of asparagus peel fibers (greater than 30%) generated more extendible foam trays.	[57]
Sugarcane bagasse	Cellulose nanocrystals	Acid hydrolysis	2, 5, and 8%	Whey protein isolate	Filler	Film	Solution casting	Film with 8% of cellulose nanocrystals provided appropriate mechanical properties and barrier properties.	[60]
Woody residues from tomato, pepper, and eggplant	Lignocellulose nanofibers	Mechanical and TEMPO mediated oxidation pre-treatment	2, 5, and 7%	Poly (vinyl alcohol)	Reinforcement	Film	Solution casting	Films reinforced with 7% lignocellulose nanofibers showed optimal results for tensile strength, Young’s modulus, and elongation at break. At 7% lignocellulose nanofibers, antioxidant activity increased between 90.9% and 191.8%, depending on the raw material and the pre-treatment used to obtain the different lignocellulose nanofibers.	[64]
Sweet potato residues	Starch and cellulose	Hot-air drying and ground into flour	5, 10, 20, 30, and 40%	Poly(3-hydroxybutyrate-co-3-hydroxyvalerate) (PHBV)	Filler	Dog bones and film	Injectionmolding and compression molding	For films at up to 10 wt% of residues, tensile strength remained over 30 MPa with strain over 3.2%.	[58]

### 3.3. Use of Animal Waste in Packaging

In the animal processing industry, feathers, skin, shells, or other body parts that are not included in the process are considered by-products that researchers have recently attempted to utilize in need of a clean industry concept. Animal wastes, including feathers, non-meat items, and eggshells, have been used to improve food security. Agro-waste from animal resources can serve as sustainable alternative food packaging and containers (Table 4). Animal hides have been used for shelters, clothing, and food packaging since primitive times [76].

Feathers are mostly a by-product from the poultry industry, which are wasted without pretreatment and create difficulty in waste management. In addition, they are 91% protein, 1% lipids, and 8% water, which potentially can be used in polymer-based materials [77]. Feathers are suitable to process under extrusion. McGauran et al. (2021) [78] found the screw speed of the extruder had a significant effect on the properties of a feather-based polymer, where a lower screw speed (≤200 rpm) was unsuccessfully applied. Screw speed can reduce the shearing effect, resulting in a larger powder size; moreover, a low flow rate leads to a longer hold time inside the process, which can dry the material inside, however, the feather-based polymer required being melted and soft to be processed. The process cycle of feather-based polymers affected degradation temperature and softening temperature. The feather pellets produced in the first cycle had a higher degradation temperature (206 °C) compared to the raw feather polymer material (168 °C), which was related to an increased softening temperature. Furthermore, the addition of 40% glycerol as a plasticizer in the feather-based film improved the elongation at break (57%) compared to 40% propylene glycol plasticizer (15%), which described the flexibility of the film. Feather protein film originating from chicken waste was applied as active packaging by incorporating gelatin and clove essential oil as a bioactive compound [77]. They found that this active film was effective in inhibiting *Escherichia coli* and *Listeria monocytogenes* by 1.41 and 1.34 log CFU/g, respectively, for smoked salmon. There was also an improvement in elongation at break, tensile strength, and water barrier properties compared to chicken feather protein without clove essential oil. Poultry feathers are dominated by keratin protein (80%) at the dry weight [78]. However, the presence of keratin crosslinked polymers with disulfide bonds between them causes difficulty in the extraction process. The extraction process required disulfide breaking under heating. Keratin contributed to reduced water solubility and mechanical property improvement of turmeric starch in a keratin–starch composite film due to the intermolecular bond between the hydroxyl group of starch and carboxyl and the amino group of keratins [79]. Barone et al. (2007) [80] reported that keratin degraded in 30 days, confirmed by 24% of carbon content metabolized under three-month-old compost inoculum.

Another animal body part that can be wasted is the skin, which has a high content of gelatin that can be utilized in a polymer-based material. Gelatin is extracted from animal skin via chemical extraction using an acid-based solvent such as sodium hydroxide, sulfuric acid, or citric acid [81,82]. Jusoh et al. (2022) [81] found the incorporation of virgin coconut oil improved water barrier properties and elongation at break, meanwhile reducing the tensile strength of chicken skin gelatin, which also contributed to the increased hydrophobicity of gelatin film with the addition of virgin coconut oil, confirmed by the presence of C=O stretching vibration of aldehyde or ester carbonyl groups. Furthermore, the addition of virgin coconut oil to chicken skin gelatin interrupted the protein–protein chain leading to structural relaxation and reducing the glass transition temperature. The incorporation of spent coffee ground extract (SCGE) in bigeye tuna skin gelatin improved tensile strength and Young’s modulus values due to the cross-linking between gelatin molecules and the phenolic compounds of SCGE and causing slight displacement confirmed by N-H bands between 3250 cm^−1^ and 3600 cm^−1^ that became wider [82]. Carp skin gelatin was also successfully incorporated with antioxidant peptide furcellaran and applied to perishable Atlantic mackerel products [83]; they found that this packaging system effectively prolonged the shelf life of Atlantic mackerel products up to 2 days.

**Figure 4:**
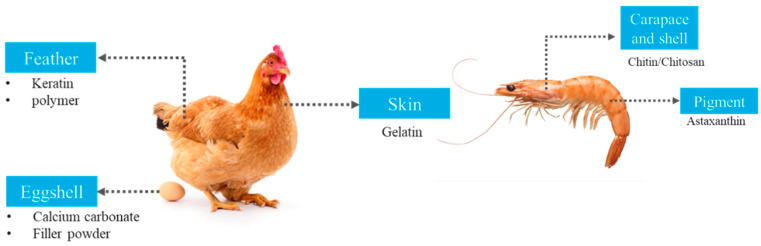
By-products from animal processing and their utilization.

Some living organisms have a shell to cover their body, influenced by living behavior or to protect egg cells in mammals. This shell is rich in organic compounds such as calcium carbonate, protein, polysaccharides, and chitin [84]. Calcium carbonate is a common organic filler used in material development. Mousavi et al. (2017) [85] studied the effect of oak shell, potassium sorbate, and eggshell nanoparticles in polyethylene terephthalate/acryl butadiene styrene copolymer for food packaging applications. Results showed improvement in mechanical properties as well as oxygen permeability compared to the neat films. Kaewtatip et al. (2018) [84] reported that starch-based foam with added eggshell filler had low density (0.2056 g/ccm^3^) with narrow cell distribution due to the nucleating agent of the eggshell filler, which contains less protein and can help the growth of steam bubbles and prevent their collapse during the baking process. Moreover, the organic compounds in the eggshell filler acted as coupling agents that increased the adhesion between starch and filler, resulting in a higher Izod impact strength value (167 J/m^2^). Shrimp shell filler addition to starch-based foam was reported to reduce Izod impact strength and create higher density due to the high protein content in the filler causing agglomeration and leading to a weakness of the bonding between materials. Furthermore, the eggshell filler also improved the closed-cell content and porosity of polyurethane foam [86]. Shells found in many aquatic biota, such as clams and crustaceans, have a variety of organic compounds such as hydroxyapatite, chitin, or calcium carbonate. Wu et al. (2020) [87] reported that PLA film incorporated with freshwater clam shell powder effectively inhibits *Escherichia coli* and *Staphylococcus aureus* with radii of 0.41 cm and 0.34 cm, respectively, due to the presence of CaO in the clamshell powder. However, the clamshell powder addition reduced tensile strength and elongation at failure of the PLA film due to aggregation of the filler. Crustaceans have a unique metabolism in their shell or carapace, which contains chitin and astaxanthin pigment. Xu et al. (2020) [88] incorporated the astaxanthin from shrimp carapaces into a gelatin/chitosan film. They found that 15% astaxanthin had antioxidant activity, up to 90% of DPPH radical scavenging activity. Moreover, astaxanthin derived from shrimp shells increased the hydrophobicity and water vapor permeability of the gelatin/chitosan film. The shrimp carapace and exoskeleton also contain a high amount of chitin. The extraction process includes demineralization using lactic acid and enzymatic deproteination, followed by a deacetylation process to convert chitin to chitosan [89]. Chitosan is useful in biodegradable packaging material that has antimicrobial antioxidant activity. Arancibia et al. (2015) [89] reported that the incorporation of chitosan and protein concentrate had a higher ABTS value (13.8 mg Vit C eq/g) compared to chitosan only (6.85 mg Vit C eq/g). Moreover, protein concentrate improved the tensile strength, puncture force, and Young’s modulus of chitosan film. Al-Ali et al. (2021) [90] synthesized edible packaging from shrimp shell chitosan containing ginger oil as an active agent. Results revealed that the films had good tensile properties and antioxidant characteristics and were potential candidates for food packaging applications [90]. Dasumiati et al. (2019) [91] developed a bioplastic from cassava peel agro-waste, with shrimp shells used as reinforcement. The films had good tensile properties and could be used as direct-wrap packages for sausages. Qian et al. (2022) [92] produced chitin from liquefied shrimp shells and polyvinyl alcohol as a base polymer matrix film to develop active packaging incorporating β-cyclodextrin/cinnamaldehyde. The β-cyclodextrin served as an encapsulating agent to delay the release of cinnamaldehyde and thereby enhance the antimicrobial activity of the film for a longer period. Real-time packaging studies showed that the films extended the shelf life of cherry tomatoes up to 10 days. These findings indicated that waste derived from animal-based agro-waste can be used as fillers and packaging matrices for sustainable packaging development. Rather et al. (2022) [93] purposed to extend the shelf life of cherry tomatoes; they created an edible coating made from lotus stem starch and gelatin from poultry waste. Gelatin from chicken feet and starch from leftover lotus stems were recovered at rates of 14.5% and 9.20%, respectively, and combined in varying amounts to create the coating substance. Chungsiriporn et al. (2022) [94] made fibrous packaging paper from oil palm fruit fiber and evaluated the effects of a beeswax–chitosan solution coating and alkali pulping procedure on the paper. The influences on the coated palm fiber base paper’s physical, mechanical, and biodegradable characteristics were also identified as potential inducements to adopt a novel kind of fibrous packaging base paper in the future. Figure 4 shows animal by-products have potential to be utilized in the packaging field, with the application and properties depending on the organic components in the waste animal body parts.

**Table 4:** Packaging developments utilizing animal waste products.

Waste Source	Components	Extraction	Contents of Waste in Packaging	Polymer Blend	Role of Waste in Packaging	Packaging Form	Production Process	Major Findings	Ref.
Chicken skin	Gelatin	Chemical extraction using sodium hydroxide	100 (*w*/*w*)	-	Matrix	Film	Solution casting	Tensile strength and glass transition temperature decreased by increasing virgin coconut oil concentration, while elongation at break and antioxidant capacity improved.	[81]
Poultry feathers	Powder	Milling and sieving	625 g	-	Matrix	Film	Compression molding	Feather/propylene glycol at ratio 30/70 exhibited optimal mechanical properties.	[78]
Chicken feathers	Keratin	Chemical extraction using sodium hydroxide	30:0, 30:1, 30:3 and 30:5 (starch:keratin *v*/*v*).	Turmeric starch	Filler	Film	Solution casting	Improved mechanical properties and water stability. Degraded by 24% within 12 days.	[79]
Shrimp and crab shell	Protein hydrolysate	Enzymatic extraction (bromelain and alcalase)	1.3- 6%	Chitosan and gelatin	Antioxidant and antimicrobial agent	Film	Casting solution	Protein hydrolysate increased the hydrophilicity of the film but decreased tensile properties.	[95]
Bigeye tuna skin	Gelatin	Chemical extraction using NaOH	5%	Spent coffee ground extract (SCGE)	Matrix	Film	Solvent casting	Increasing SCGE concentration improved solubility, moisture content, water vapor permeability, and transparency of the film.	[82]
Common carp skin	Gelatin hydrolysate	Extraction using NaCl, NaOH, H_2_SO_4_, and C_6_H_8_O_7_.	5 g	Furcellaran	Matrix	Film	Solution casting	Gelatin film containing furcellaran effectively delayed lipid oxidation and inhibited microbial growth on Atlantic mackerel.	[83]
Shrimp waste (head, shells of cephalothorax, and tails)	Astaxanthin	Extraction using ethanol	15 μg/mL	Chitosan, gelatin	Antioxidant and antimicrobial compound	Film	Solution casting	Astaxanthin effectively retarded the oxidation of corn oil and showed good microbial resistance.	[88]
Freshwater clam shells	Nanofiber	Thermal calcination process	10 g in 200 mL dicumyl peroxide	PLA and fish gelatin	Antibacterial	Fiber	Electrospinning	Good antimicrobial properties. Excellent tensile strength.	[87]
Chicken egg shells	Powder	Grinding and sieving	20 parts by weight	Polyurethane	Filler	Rigid foam	Compression molding	Filler improved the dimensional stability of rigid foam and limited microbial growth. Filler decreased the glass transition temperature.	[86]
Chicken feathers	Fiber	Shredding	1:10	Polyethylene with a polypropylene core	Matrix	Non-woven feather fiber composite	Air-laid process	Comparable thermal performance to expanded polystyrene in terms of time–temperature profile of meat substitute with coolants inside the packaging.	[96]
Eggshells	Silver-doped hydroxyapatite	Sonication	0.5–4%	Polyurethane and algae oil	Antibacterial filler	Coating film	Coating bar applicator	Coating material improved hydrophobicity, adhesion, and anticorrosive and antimicrobial properties.	[97]
Egg and shrimp shells	Powder	Grinding	0–20 wt%	Native cassava starch, glycerol	Filler	Foam	Compression molding	Eggshell addition improved Izod impact strength and reduced density, with narrow cell distribution of the starch foam. Degradation temperature decreased with filler addition.	[84]
Eggshell membranes	Gelatin	Extraction with alkali solution	25–100%	Chitosan	Matrix	Film	Solution casting	Decreased water solubility and water vapor permeability compared to single material film. The microstructure showed that gelatin and chitosan were totally miscible. Chitosan addition improved elongation at break of the composite film.	[98]
Eggshells	Powder	Milling and sieving	5, 10, 15, 20, 25 parts by weight	Polyurethane	Filler	Rigid foam	Compression molding	Eggshell filler increased apparent density and compressive strength in the orientation parallel to the foam growth direction. The degree of phase separation of foam material also increased.	[99]
Shrimp cephalothoraxes and exoskeletons	Chitosan	Organic extraction using acetone and ethanol	1:1	Lactic acid and protein concentrate	Antioxidant and antimicrobial matrix	Film	Casting solution	Improved tensile strength, Young’s modulus, elongation at break, and puncture force but reduced puncture deformation. Good antimicrobial resistance.	[89]
White chicken feathers	Protein	Chemical extraction with NaOH	5 g	Gelatin (0.5, 1, 1.5, 2 g), clove essential oil/cinnamaldehyde (0.5, 1, 1.5 g),	Matrix	Film	Solution casting	Mechanical properties increased. Films with clove oil had strong antimicrobial properties.	[77]
Feathers	Keratin	Ground and sieving	50%	Glycerol:DI-H_2_O:Na_2_SO_3_30:18.5:1.5 wt%	Matrix	Thin sheet	Extrusion	24% carbon in the sheet was degraded within 30 days.	[80]
Chicken feet wasteLotus stem	GelatinStarch powder	NaOH and HCl	4 g	Lotus steam starch	Matrix	Film	Coating	The edible coating extended the shelf life of cherry tomatoes up to 15 days.	[93]

## 4. Conclusions and Future Perspectives

Agro-waste management is now a serious concern as the global population grows. Valorization of these wastes into useful products will both benefit the environment and save a large amount of revenue. New developments in eco-friendly food packaging have increased with growing consumer demand. Food agro-waste from many diverse sources can be extracted and purified to produce food packaging components. Wastes originating from crop and processing residues as well as animal by-products have been extensively investigated. Extraction methods, processing conditions, and blending materials have a significant effect on the properties of the composite materials, including mechanical, barrier, and thermal properties. Materials such as cellulose and eggshell powder have been successfully used as fillers or reinforcements in some rigid packaging to improve their mechanical properties, while feather keratin has been developed as a new material. To achieve valorization, innovative packaging strategies must be promoted by governments and representative authorities. Agricultural industries generate huge volumes of waste and by-products during production, handling, and product processing. Disposal of these wastes is now a serious financial and ecological concern with detrimental environmental effects. Therefore, alternative methods of recycling and reprocessing these wastes is a significant research target. Development of composite materials as low-cost packaging alternatives with enhanced properties is now urgently required for large-scale, economically affordable production. Process optimization of these materials and safety concerns about potential migration of substances must also be further explored. This review indicated that various sources of agro-waste can be fabricated into packaging materials and fillers as reinforcements or barrier improvements. Agro-waste requires extraction and purification before formulation as packaging material. The collective method, storage, preservation, and treatment of the waste before processing will be a huge challenge for the research and industrial sectors. Legislative steps must be urgently taken for large-scale utilization of agro-waste to benefit the environment through sustainability.

## Figures and Tables

**Table 1:** Major component percentages of common agro-residues.

Agro-Waste	Lignin (%)	α Cellulose (%)	Hemicellulose (%)
Banana peel	11 ± 1.12	9 ± 0.08	3 ± 0.55
Cassava peel	39 ± 0.34	8 ± 0.52	25 ± 0.41
Cornstalks	30 ± 1.19	15 ± 0.67	33 ± 2.63
Empty fruit bunches	25.79	19.30	25.55
Finger millet hulls	25 ± 0.73	32 ± 1.38	4 ± 1.6
Groundnut husks	36 ± 1. 41	4 20 ± 0.88	25 ± 1.03
Rice	31.97	31.10	18.35
Rice husks	18.82	41.05	17.63
Rice straw	35 ± 1.31	27 ± 1.97	16 ± 2.01
Sawdust	44 ± 1.77	17 ± 0.14	21 ± 1.92
Shea nut cake	26 ± 0.26	9 ± 0.33	21 ± 0.09
Sorghum hulls	39 ± 0.82	35± 0.07	4 ± 0.11
Soybean hulls	35 ± 0.12	16 ± 0.21	4 ± 0.19
Sugarcane bagasse	45 ± 0.52	26 ± 0.34	19 ± 0.13

## References

[1] Corrado S., Caldeira C., Eriksson M., Hanssen O.J., Hauser H.E., van Holsteijn F., Liu G., Östergren K., Parry A., Secondi L. (2019). Food waste accounting methodologies: Challenges, opportunities, and further advancements. Glob. Food Secur..

[2] Shafiee-Jood M., Cai X. (2016). Reducing Food Loss and Waste to Enhance Food Security and Environmental Sustainability. Environ. Sci. Technol..

[3] Chaboud G., Daviron B. (2017). Food losses and waste: Navigating the inconsistencies. Glob. Food Secur..

[4] Ishangulyyev R., Kim S., Lee S.H. (2019). Understanding Food Loss and Waste—Why Are We Losing and Wasting Food?. Foods.

[5] Ciccullo F., Cagliano R., Bartezzaghi G., Perego A. (2020). Implementing the circular economy paradigm in the agri-food supply chain: The role of food waste prevention technologies. Resour. Conserv. Recycl..

[6] Spang E.S., Moreno L.C., Pace S.A., Achmon Y., Donis-Gonzalez I., Gosliner W.A., Jablonski-Sheffield M.P., Momin M.A., Quested T.E., Winans K.S. (2019). Food Loss and Waste: Measurement, Drivers, and Solutions. Annu. Rev. Environ. Resour..

[7] Wohner B., Pauer E., Heinrich V., Tacker M. (2019). Packaging-Related Food Losses and Waste: An Overview of Drivers and Issues. Sustainability.

[8] Laorenza Y., Chonhenchob V., Bumbudsanpharoke N., Jittanit W., Sae-Tan S., Rachtanapun C., Chanput W.P., Charoensiddhi S., Srisa A., Promhuad K. (2022). Polymeric Packaging Applications for Seafood Products: Packaging-Deterioration Relevance, Technology and Trends. Polymers.

[9] Promsorn J., Harnkarnsujarit N. (2022). Pyrogallol loaded thermoplastic cassava starch based films as bio-based oxygen scavengers. Ind. Crop. Prod..

[10] Promhuad K., Bumbudsanpharoke N., Wadaugsorn K., Sonchaeng U., Harnkarnsujarit N. (2022). Maltol-Incorporated Acetylated Cassava Starch Films for Shelf-Life-Extension Packaging of Bakery Products. Polymers.

[11] San H., Laorenza Y., Behzadfar E., Sonchaeng U., Wadaugsorn K., Sodsai J., Kaewpetch T., Promhuad K., Srisa A., Wongphan P. (2022). Functional Polymer and Packaging Technology for Bakery Products. Polymers.

[12] Srisa A., Promhuad K., San H., Laorenza Y., Wongphan P., Wadaugsorn K., Sodsai J., Kaewpetch T., Tansin K., Harnkarnsujarit N. (2022). Antibacterial, Antifungal and Antiviral Polymeric Food Packaging in Post-COVID-19 Era. Polymers.

[13] Promhuad K., Srisa A., San H., Laorenza Y., Wongphan P., Sodsai J., Tansin K., Phromphen P., Chartvivatpornchai N., Ngoenchai P. (2022). Applications of Hemp Polymers and Extracts in Food, Textile and Packaging: A Review. Polymers.

[14] Sharma R., Ghoshal G. (2018). Emerging trends in food packaging. Nutr. Food Sci..

[15] Sonchaeng U., Promsorn J., Bumbudsanpharoke N., Chonhenchob V., Sablani S.S., Harnkarnsujarit N. (2022). Polyesters Incorporating Gallic Acid as Oxygen Scavenger in Biodegradable Packaging. Polymers.

[16] Tummala R.R. Packaging: Past, present and future. Proceedings of the 2005 6th International Conference on Electronic Packaging Technology.

[17] Jain R., Tiwari A. (2015). Biosynthesis of planet friendly bioplastics using renewable carbon source. J. Environ. Health Sci. Eng..

[18] Kaewpetch T., Pratummang A., Suwarak S., Wongphan P., Promhuad K., Leelaphiwat P., Bumbudsanpharoke N., Lorenzo J.M., Harnkarnsujarit N. (2023). Ylang-ylang (Cananga odorata) essential oils with flora odorants enhanced active function of biodegradable polyester films produced by extrusion. Food Biosci..

[19] Qian M., Liu D., Zhang X., Yin Z., Ismail B.B., Ye X., Guo M. (2021). A review of active packaging in bakery products: Applications and future trends. Trends Food Sci. Technol..

[20] Phothisarattana D., Harnkarnsujarit N. (2022). Migration, aggregations and thermal degradation behaviors of TiO_2_ and ZnO incorporated PBAT/TPS nanocomposite blown films. Food Packag. Shelf Life.

[21] Riaz A., Lei S., Akhtar H.M.S., Wan P., Chen D., Jabbar S., Abid M., Hashim M.M., Zeng X. (2018). Preparation and characterization of chitosan-based antimicrobial active food packaging film incorporated with apple peel polyphenols. Int. J. Biol. Macromol..

[22] Madurwar M.V., Ralegaonkar R.V., Mandavgane S.A. (2013). Application of agro-waste for sustainable construction materials: A review. Constr. Build. Mater..

[23] Yan B., Feng L., Zheng J., Zhang Q., Jiang S., Zhang C., Ding Y., Han J., Chen W., He S. (2022). High performance supercapacitors based on wood-derived thick carbon electrodes synthesized *via* green activation process. Inorg. Chem. Front..

[24] Feng L., Yan B., Zheng J., Chen J., Wei R., Jiang S., Yang W., Zhang Q., He S. (2022). Soybean protein-derived N, O co-doped porous carbon sheets for supercapacitor applications. New J. Chem..

[25] Obey G., Adelaide M., Ramaraj R. (2022). Biochar derived from non-customized matamba fruit shell as an adsorbent for wastewater treatment. J. Bioresour. Bioprod..

[26] Mitura K., Kornacka J., Kopczyńska E., Kalisz J., Czerwińska E., Affeltowicz M., Kaczorowski W., Kolesińska B., Frączyk J., Bakalova T. (2021). Active Carbon-Based Nanomaterials in Food Packaging. Coatings.

[27] Dilucia F., LaCivita V., Conte A., Del Nobile M.A. (2020). Sustainable Use of Fruit and Vegetable By-Products to Enhance Food Packaging Performance. Foods.

[28] Sharma V., Tsai M.-L., Nargotra P., Chen C.-W., Kuo C.-H., Sun P.-P., Dong C.-D. (2022). Agro-Industrial Food Waste as a Low-Cost Substrate for Sustainable Production of Industrial Enzymes: A Critical Review. Catalysts.

[29] Sadh P.K., Duhan S., Duhan J.S. (2018). Agro-industrial wastes and their utilization using solid state fermentation: A review. Bioresour. Bioprocess..

[30] Bhuimbar M.V., Bhagwat P.K., Dandge P.B. (2019). Extraction and characterization of acid soluble collagen from fish waste: Development of collagen-chitosan blend as food packaging film. J. Environ. Chem. Eng..

[31] Hussain C., Singh S., Goswami L. (2021). Emerging Trends to Approaching Zero Waste: Environmental and Social Perspectives.

[32] Resource Conservation and Recovery Act (RCRA) Code of Federal Regulations, Identification and listing of hazardous waste, 40 (1997): Section 261.4 (Exclusion, Materials which are not solid waste). 32. Regulatory Exclusions and Alternative Standards for the Recycling of Materials, Solid Wastes and Hazardous Wastes.” US EPA, 26 Jan. 2023. www.epa.gov/hw/regulatory-exclusions-and-alternative-standards-recycling-materials-solid-wastes-and-hazardous.

[33] Risdianto H., Sofianti E., Suhardi S.H., Setiadi T. (2012). Optimisation of Laccase Production using White Rot Fungi and Agriculture Wastes in Solid State Fermentation. ITB J. Eng. Sci..

[34] Salihu A., Abbas O., Sallau A.B., Alam Z. (2015). Agricultural residues for cellulolytic enzyme production by Aspergillus niger: Effects of pretreatment. 3 Biotech.

[35] Wang J., Euring M., Ostendorf K., Zhang K. (2022). Biobased materials for food packaging. J. Bioresour. Bioprod..

[36] Oyeoka H.C., Ewulonu C.M., Nwuzor I.C., Obele C.M., Nwabanne J.T. (2021). Packaging and degradability properties of polyvinyl alcohol/gelatin nanocomposite films filled water hyacinth cellulose nanocrystals. J. Bioresour. Bioprod..

[37] Kora A.J. (2019). Leaves as dining plates, food wraps and food packing material: Importance of renewable resources in Indian culture. Bull. Natl. Res. Cent..

[38] Manan S., Ullah M.W., Ul-Islam M., Atta O.M., Yang G. (2021). Synthesis and applications of fungal mycelium-based advanced functional materials. J. Bioresour. Bioprod..

[39] Dissanayake D.G.K., Weerasinghe D., Perera T.D.R., Bandara M.M.A.L., Thathsara S.K.T., Perera S. (2021). A Sustainable Transparent Packaging Material from the Arecanut Leaf Sheath. Waste Biomass- Valorization.

[40] Kaya M., Khadem S., Cakmak Y.S., Mujtaba M., Ilk S., Akyuz L., Salaberria A.M., Labidi J., Abdulqadir A.H., Deligöz E. (2018). Antioxidative and antimicrobial edible chitosan films blended with stem, leaf and seed extracts of Pistacia terebinthus for active food packaging. RSC Adv..

[41] Campos D., Piccirillo C., Pullar R.C., Castro P.M., Pintado M.M. (2014). Characterization and antimicrobial properties of food packaging methylcellulose films containing stem extract of Ginja cherry. J. Sci. Food Agric..

[42] Fontes-Candia C., Erboz E., Martínez-Abad A., López-Rubio A., Martínez-Sanz M. (2019). Superabsorbent food packaging bioactive cellulose-based aerogels from Arundo donax waste biomass. Food Hydrocoll..

[43] Berthet M.A., Angellier-Coussy H., Chea V., Guillard V., Gastaldi E., Gontard N. (2015). Sustainable food packaging: Valorising wheat straw fibres for tuning PHBV-based composites properties. Compos. Part A Appl. Sci. Manuf..

[44] Elhussieny A., Faisal M., D’Angelo G., Aboulkhair N.T., Everitt N.M., Fahim I.S. (2020). Valorisation of shrimp and rice straw waste into food packaging applications. Ain Shams Eng. J..

[45] Benito-González I., López-Rubio A., Martínez-Sanz M.M. (2019). High-performance starch biocomposites with cellulose from waste biomass: Film properties and retrogradation behaviour. Carbohydr. Polym..

[46] Kumar A., Ramakanth D., Akhila K., Gaikwad K.K. (2023). Influence of halloysite nanotubes/microfibrillated cellulose on pine leaves waste based ethylene scavenging composite paper for food packaging applications. Appl. Clay Sci..

[47] Tran T.N., Lim K.T., Fiorentini F., Athanassiou A. (2022). Antioxidant and Biocompatible CO _2_ -Based Biocomposites from Vegetable Wastes for Active Food Packaging. Adv. Sustain. Syst..

[48] Olawuyi I.F., Lee W.Y. (2022). Development and Characterization of Biocomposite Films Based on Polysaccharides Derived from Okra Plant Waste for Food Packaging Application. Polymers.

[49] Lima E.M.B., Middea A., Neumann R., Thiré R.M.D.S.M., Pereira J.F., de Freitas S.C., Penteado M.S., Lima A.M., Minguita A.P.D.S., Mattos M.D.C. (2021). Biocomposites of PLA and Mango Seed Waste: Potential Material for Food Packaging and a Technological Alternative to Reduce Environmental Impact. Starch.

[50] Thivya P., Bhosale Y.K., Anandakumar S., Hema V., Sinija V.R. (2021). Exploring the Effective Utilization of Shallot Stalk Waste and Tamarind Seed for Packaging Film Preparation. Waste Biomass- Valorization.

[51] Rincón E., Espinosa E., García-Domínguez M., Balu A., Vilaplana F., Serrano L., Jiménez-Quero A. (2021). Bioactive pectic polysaccharides from bay tree pruning waste: Sequential subcritical water extraction and application in active food packaging. Carbohydr. Polym..

[52] Bilo F., Pandini S., Sartore L., Depero L.E., Gargiulo G., Bonassi A., Federici S., Bontempi E. (2018). A sustainable bioplastic obtained from rice straw. J. Clean. Prod..

[53] Kasaai M.R., Moosavi A. (2017). Treatment of Kraft paper with citrus wastes for food packaging applications: Water and oxygen barrier properties improvement. Food Packag. Shelf Life.

[54] de Oliveira A.L.M., Bento J.A.C., Fidelis M.C., Dias M.C., de Barros H.E.A., Natarelli C.V.L., Lago R.C.D., Barbosa J.W., Ossani P.C., Caliari M. (2023). Effect of pine, eucalyptus, and corn straw nanofibers on the structural properties of rice flour-based biodegradable films. Ind. Crop. Prod..

[55] Riaz A., Lagnika C., Luo H., Dai Z., Nie M., Hashim M.M., Liu C., Song J., Li D. (2020). Chitosan-based biodegradable active food packaging film containing Chinese chive (Allium tuberosum) root extract for food application. Int. J. Biol. Macromol..

[56] Melendez-Rodriguez B., Torres-Giner S., Aldureid A., Cabedo L., Lagaron J.M. (2019). Reactive Melt Mixing of Poly(3-Hydroxybutyrate)/Rice Husk Flour Composites with Purified Biosustainably Produced Poly(3-Hydroxybutyrate-*co*-3-Hydroxyvalerate). Materials.

[57] Cruz-Tirado J.P., Vejarano R., Tapia-Blácido D.R., Angelats-Silva L.M., Siche R. (2019). The addition of sugarcane bagasse and asparagus peel enhances the properties of sweet potato starch foams. Packag. Technol. Sci..

[58] Vannini M., Marchese P., Sisti L., Saccani A., Mu T., Sun H., Celli A. (2021). Integrated Efforts for the Valorization of Sweet Potato By-Products within a Circular Economy Concept: Biocomposites for Packaging Applications Close the Loop. Polymers.

[59] Tkaczewska J., Jamróz E., Zając M., Guzik P., Gedif H.D., Turek K., Kopeć M. (2023). Antioxidant edible double-layered film based on waste from soybean production as a vegan active packaging for perishable food products. Food Chem..

[60] Sukyai P., Anongjanya P., Bunyahwuthakul N., Kongsin K., Harnkarnsujarit N., Sukatta U., Sothornvit R., Chollakup R. (2018). Effect of cellulose nanocrystals from sugarcane bagasse on whey protein isolate-based films. Food Res. Int..

[61] Azmin S.N.H.M., Hayat N.A.B.M., Nor M.S.M. (2020). Development and characterization of food packaging bioplastic film from cocoa pod husk cellulose incorporated with sugarcane bagasse fibre. J. Bioresour. Bioprod..

[62] Sasimowski E., Majewski Ł., Grochowicz M. (2021). Artificial Ageing, Chemical Resistance, and Biodegradation of Biocomposites from Poly(Butylene Succinate) and Wheat Bran. Materials.

[63] Abotbina W., Sapuan S.M., Ilyas R.A., Sultan M.T.H., Alkbir M.F.M. (2022). Preparation and Characterization of Black Seed/Cassava Bagasse Fiber-Reinforced Cornstarch-Based Hybrid Composites. Sustainability.

[64] Bascón-Villegas I., Sánchez-Gutiérrez M., Pérez-Rodríguez F., Espinosa E., Rodríguez A. (2021). Lignocellulose Nanofibre Obtained from Agricultural Wastes of Tomato, Pepper and Eggplants Improves the Performance of Films of Polyvinyl Alcohol (PVA) for Food Packaging. Foods.

[65] Rodríguez-Félix F., Corte-Tarazón J.A., Rochín-Wong S., Fernández-Quiroz J.D., Garzón-García A.M., Santos-Sauceda I., Plascencia-Martínez D.F., Chan-Chan L.H., Vásquez-López C., Barreras-Urbina C.G. (2022). Physicochemical, structural, mechanical and antioxidant properties of zein films incorporated with no-ultrafiltered and ultrafiltered betalains extract from the beetroot (*Beta vulgaris*) bagasse with potential application as active food packaging. J. Food Eng..

[66] Moustafa H., El-Wakil A.E.-A.A., Nour M.T., Youssef A.M. (2020). Kenaf fibre treatment and its impact on the static, dynamic, hydrophobicity and barrier properties of sustainable polystyrene biocomposites. RSC Adv..

[67] Bascón-Villegas I., Pereira M., Espinosa E., Sánchez-Gutiérrez M., Rodríguez A., Pérez-Rodríguez F. (2022). A new eco-friendly packaging system incorporating lignocellulose nanofibres from agri-food residues applied to fresh-cut lettuce. J. Clean. Prod..

[68] Kumar G.M., Kumar R.P. (2018). Characterization of pine needle ash particulates reinforced surface composite fabricated by friction stir process. Mater. Res. Express.

[69] Menzel C., González-Martínez C., Vilaplana F., Diretto G., Chiralt A. (2020). Incorporation of natural antioxidants from rice straw into renewable starch films. Int. J. Biol. Macromol..

[70] Freitas P.A., González-Martínez C., Chiralt A. (2023). Using rice straw fractions to develop reinforced, active PLA-starch bilayers for meat preservation. Food Chem..

[71] Ravindran R., Jaiswal A.K. (2016). A comprehensive review on pre-treatment strategy for lignocellulosic food industry waste: Challenges and opportunities. Bioresour. Technol..

[72] Travalini A.P., Lamsal B., Magalhães W.L.E., Demiate I.M. (2019). Cassava starch films reinforced with lignocellulose nanofibers from cassava bagasse. Int. J. Biol. Macromol..

[73] Ibrahim M.I.J., Sapuan S.M., Zainudin E.S., Zuhri M.Y.M. (2020). Preparation and characterization of cornhusk/sugar palm fiber reinforced Cornstarch-based hybrid composites. J. Mater. Res. Technol..

[74] Zhou Y., Fan M., Chen L. (2016). Interface and bonding mechanisms of plant fibre composites: An overview. Compos. Part B: Eng..

[75] Cardoso T., Esmerino L.A., Bolanho B.C., Demiate I.M., Danesi E.D.G. (2019). Technological viability of biobased films formulated with cassava by-product and *Spirulina platensis*. J. Food Process. Eng..

[76] Jayathilakan K., Sultana K., Radhakrishna K., Bawa A.S. (2012). Utilization of byproducts and waste materials from meat, poultry and fish processing industries: A review. J. Food Sci. Technol..

[77] Song N.-B., Lee J.-H., Al Mijan M., Bin Song K. (2014). Development of a chicken feather protein film containing clove oil and its application in smoked salmon packaging. Lwt.

[78] McGauran T., Harris M., Dunne N., Smyth B.M., Cunningham E. (2021). Development and optimisation of extruded bio-based polymers from poultry feathers. Eur. Polym. J..

[79] Oluba O.M., Osayame E., Shoyombo A.O. (2021). Production and characterization of keratin-starch bio-composite film from chicken feather waste and turmeric starch. Biocatal. Agric. Biotechnol..

[80] Barone J.R., Arikan O. (2007). Composting and biodegradation of thermally processed feather keratin polymer. Polym. Degrad. Stab..

[81] Jusoh N., Isa M., Sarbon N. (2022). Physical, mechanical and antioxidant properties of chicken skin gelatin films incorporated with virgin coconut oil. Biocatal. Agric. Biotechnol..

[82] Getachew A.T., Ahmad R., Park J.-S., Chun B.-S. (2021). Fish skin gelatin based packaging films functionalized by subcritical water extract from spent coffee ground. Food Packag. Shelf Life.

[83] Tkaczewska J., Kulawik P., Jamróz E., Guzik P., Zając M., Szymkowiak A., Turek K. (2021). One- and double-layered furcellaran/carp skin gelatin hydrolysate film system with antioxidant peptide as an innovative packaging for perishable foods products. Food Chem..

[84] Kaewtatip K., Chiarathanakrit C., Riyajan S.-A. (2018). The effects of egg shell and shrimp shell on the properties of baked starch foam. Powder Technol..

[85] Mousavi S.M., Hashemi S.A., Amani A.M., Saed H., Jahandideh S., Mojoudi F. (2017). Polyethylene Terephthalate/Acryl Butadiene Styrene Copolymer Incorporated with Oak Shell, Potassium Sorbate and Egg Shell Nanoparticles for Food Packaging Applications: Control of Bacteria Growth, Physical and Mechanical Properties. Polym. Renew. Resour..

[86] Leszczyńska M., Ryszkowska J., Szczepkowski L., Kurańska M., Prociak A., Leszczyński M.K., Gloc M., Antos-Bielska M., Mizera K. (2020). Cooperative effect of rapeseed oil-based polyol and egg shells on the structure and properties of rigid polyurethane foams. Polym. Test..

[87] Wu D.-Y., Wang S.-S., Wu C.-S. (2020). Antibacterial properties and cytocompatibility of biobased nanofibers of fish scale gelatine, modified polylactide, and freshwater clam shell. Int. J. Biol. Macromol..

[88] Xu J., Wei R., Jia Z., Song R. (2019). Characteristics and bioactive functions of chitosan/gelatin-based film incorporated with ε-polylysine and astaxanthin extracts derived from by-products of shrimp (Litopenaeus vannamei). Food Hydrocoll..

[89] Arancibia M.Y., Alemán A., López-Caballero M.E., Gómez-Guillén M.C., Montero P. (2015). Development of active films of chitosan isolated by mild extraction with added protein concentrate from shrimp waste. Food Hydrocoll..

[90] Al-Ali R.M., Al-Hilifi S.A., Rashed M.M. (2021). Fabrication, characterization, and anti-free radical performance of edible packaging-chitosan film synthesized from shrimp shell incorporated with ginger essential oil. J. Food Meas. Charact..

[91] Dasumiati, Saridewi N., Malik M. (2019). Food packaging development of bioplastic from basic waste of cassava peel (manihot uttilisima) and shrimp shell. IOP Conf. Ser. Mater. Sci. Eng..

[92] Qian Z.-J., Zhang J., Xu W.-R., Zhang Y.-C. (2022). Development of active packaging films based on liquefied shrimp shell chitin and polyvinyl alcohol containing β-cyclodextrin/cinnamaldehyde inclusion. Int. J. Biol. Macromol..

[93] Rather J.A., Makroo H.A., Showkat Q.A., Majid D., Dar B. (2022). Recovery of gelatin from poultry waste: Characteristics of the gelatin and lotus starch-based coating material and its application in shelf-life enhancement of fresh cherry tomato. Food Packag. Shelf Life.

[94] Chungsiriporn J., Khunthongkaew P., Wongnoipla Y., Sopajarn A., Karrila S., Iewkittayakorn J. (2022). Fibrous packaging paper made of oil palm fiber with beeswax-chitosan solution to improve water resistance. Ind. Crop. Prod..

[95] Hajji S., Kchaou H., Bkhairia I., Salem R.B.S.-B., Boufi S., Debeaufort F., Nasri M. (2021). Conception of active food packaging films based on crab chitosan and gelatin enriched with crustacean protein hydrolysates with improved functional and biological properties. Food Hydrocoll..

[96] Dieckmann E., Nagy B., Yiakoumetti K., Sheldrick L., Cheeseman C. (2019). Thermal insulation packaging for cold-chain deliveries made from feathers. Food Packag. Shelf Life.

[97] Patil C.K., Jirimali H.D., Paradeshi J.S., Chaudhari B.L., Gite V.V. (2018). Functional antimicrobial and anticorrosive polyurethane composite coatings from algae oil and silver doped egg shell hydroxyapatite for sustainable development. Prog. Org. Coat..

[98] Mohammadi R., Mohammadifar M.A., Rouhi M., Kariminejad M., Mortazavian A.M., Sadeghi E., Hasanvand S. (2018). Physico-mechanical and structural properties of eggshell membrane gelatin- chitosan blend edible films. Int. J. Biol. Macromol..

[99] Zieleniewska M., Leszczyński M.K., Szczepkowski L., Bryśkiewicz A., Krzyżowska M., Bień K., Ryszkowska J. (2016). Development and applicational evaluation of the rigid polyurethane foam composites with egg shell waste. Polym. Degrad. Stab..

